# Gamma band pitch responses in human auditory cortex measured with magnetoencephalography

**DOI:** 10.1016/j.neuroimage.2011.08.098

**Published:** 2012-01-16

**Authors:** William Sedley, Sundeep Teki, Sukhbinder Kumar, Tobias Overath, Gareth R. Barnes, Timothy D. Griffiths

**Affiliations:** a Auditory Group, Institute of Neuroscience, Newcastle University Medical School, Newcastle Upon Tyne, Tyne and Wear, NE2 4HH UK; b Wellcome Trust Centre for Neuroimaging, University College London, 12 Queen Square, London, WC1N 3BG UK; c Ear Institute, University College London, 332 Grays Inn Road, London, WC1X 8EE UK

**Keywords:** Pitch, Auditory, Magnetoencephalography, Gamma, Beamformer, Perception

## Abstract

We have previously used direct electrode recordings in two human subjects to identify neural correlates of the perception of pitch (Griffiths, Kumar, Sedley et al., Direct recordings of pitch responses from human auditory cortex, Curr. Biol. 22 (2010), pp. 1128–1132). The present study was carried out to assess virtual-electrode measures of pitch perception based on non-invasive magnetoencephalography (MEG). We recorded pitch responses in 13 healthy volunteers using a passive listening paradigm and the same pitch-evoking stimuli (regular interval noise; RIN) as in the previous study. Source activity was reconstructed using a beamformer approach, which was used to place virtual electrodes in auditory cortex. Time-frequency decomposition of these data revealed oscillatory responses to pitch in the gamma frequency band to occur, in Heschl's gyrus, from 60 Hz upwards. Direct comparison of these pitch responses to the previous depth electrode recordings shows a striking congruence in terms of spectrotemporal profile and anatomical distribution. These findings provide further support that auditory high gamma oscillations occur in association with RIN pitch stimuli, and validate the use of MEG to assess neural correlates of normal and abnormal pitch perception.

## Highlights

► High gamma-band correlates of pitch perception identified with MEG beamforming. ► Results correlate strongly with invasive electrode recordings of same responses. ► Validation of accuracy of MEG beamformer approach.

## Introduction

Pitch is a perceptual property of sound that has a complex relationship to the temporal and spectral properties of acoustic stimuli (de Cheveigné, 2005). Studies of animal and human cortex have examined temporal and spectral sensory representation relevant to pitch and neural correlates of the pitch percept itself. With respect to the latter, these studies have examined the possible existence of a ‘pitch centre’ in auditory cortex where neural activity corresponds to the perceived pitch irrespective of the stimulus with which it is associated, analogous to the concept of a ‘colour centre’ in the visual system (Zeki, 1980).

We focus here on electromagnetic methods for the examination of responses relevant to pitch where the millisecond temporal resolution allows such responses to be followed with precision. A number of recent functional magnetic resonance imaging (fMRI) studies (Griffiths et al., 2001; Patterson et al., 2002; Penagos et al., 2004; Hall and Plack, 2009) have also examined haemodynamic responses to pitch using the blood oxygenation-level dependent (BOLD) response, but do not allow temporally precise examination of responses to the pitch of single stimuli or transitions from control stimuli to pitch stimuli. Moreover, the studies do not allow disambiguation of time-locked responses that might be related to the stimulus property of regularity (implicated in the majority of current models of pitch perception; de Cheveigné, 2005) as opposed to induced activity that has been suggested as a neural correlate of the pitch percept itself (Griffiths et al., 2010).

Single neuron recordings in marmosets have shown pitch-related responses in certain neurons in a single area of non-primary auditory cortex (Bendor and Wang, 2005), although recordings in the ferret do not support the existence of a single pitch centre (Bizley et al., 2009; Schnupp et al., 2010). In humans, direct recording of local-field potentials from groups of neurons (Griffiths et al., 2010) has demonstrated evoked potentials in primary and adjacent non-primary auditory cortex that can be related to the temporal regularity of the stimulus. Non-invasive recording of human brain activity using magnetoencephalography (MEG; Krumbholz et al., 2003) has also shown evoked activity during the presentation of regular stimuli associated with pitch; source localisation of this activity implicates similar areas in the medial part of Heschl's gyrus (HG) to those from which the direct recordings were made (Griffiths et al., 2010).

Depth electrode recordings from human auditory cortex also allow the examination of oscillatory activity during the presentation of regular interval noise (RIN) that is not precisely time-locked to the stimulus (Griffiths et al., 2010). Such activity was present in and around the range of 80–120 Hz throughout most areas of auditory cortex sampled (including primary auditory cortex and non primary cortex in lateral HG). It was maximal in the medial and mid parts of HG, peaked between 70 and 300 ms from RIN onset, had a weaker sustained component for the duration of the RIN stimulus and, crucially, was only present when the repetition rate of the RIN was above the lower limit of pitch. The onset of a slower rate of RIN, not associated with pitch, is still a perceptually salient event, and the lack of an associated gamma response is a compelling argument for the gamma responses as a correlate of pitch perception per se rather than simply being a generic response to a changing auditory stimulus.

Sampling limitations make it very difficult to acquire depth electrode data from multiple locations in any species and work in humans is further limited by the serendipitous nature of any such experiments, in which electrode placement is determined by clinical need. In the present experiment we assessed the utility of a non-invasive MEG technique in which ‘virtual electrodes’ can be placed in any position in a patient or healthy volunteer using a beamformer approach. A problem with using non-invasive electrophysiological methods, such as electroencephalography (EEG) and MEG, to measure oscillatory activity is that the signal to noise ratio (SNR) is very low at high frequencies. The EEG/MEG spectrum is dominated by 10 Hz (alpha) activity, and power in a given spectral range is inversely proportional to its frequency. Muscle artefacts produce high-frequency activity, and even very small muscle contractions and resting-state tonic contraction that remain after artefact rejection procedures produce high frequency activity that is several orders of magnitude stronger than brain activity at equivalent frequencies (Pope et al., 2009). Beamforming is a spatial filtering technique that, when applied to EEG and MEG, allows source reconstruction of power within a given time-frequency window (or contrast between a pair of windows) at a particular location in a way that suppresses interference from other cortical and from non-brain sources (van Veen et al., 1997). The technique is ideally suited to low SNR conditions. It also allows the generation of time series data from these locations of interest or ‘virtual electrodes’. These can be analysed using time-frequency analysis methods to show power changes that are essentially undetectable at sensor level. Beamforming has often been used to analyse activity in the gamma range, typically 40–80 Hz (Adjamian et al., 2004; Hall et al., 2005; Muthukumaraswamy et al., 2009), but activity in a higher range, from around 80 Hz upwards, is less studied outside of intracranial recording techniques (Jerbi et al., 2009).

In the present study we sought to replicate the principal results of our depth electrode pitch recordings non-invasively using MEG. We thus approached the experiment with some strong a-priori hypotheses: 1) the onset of noise from silence would be associated with a brief period of increased power centred around 80–120 Hz, but covering a wide frequency range, that would cease at around 200 ms and not be associated with a power increase beyond that time, 2) the transition from noise to RIN would be associated with an increase in power centred around 80–120 Hz that would be maximal between around 70 and 300 ms from the onset of RIN. The increase in power in this band might be sustained until just after the offset of RIN, but we acknowledged that our methods might not be sensitive enough to detect this weaker, sustained activity, and 3) these observed responses should be present in medial and mid parts of HG, and might also extend to lateral HG and planum temporale (PT). We took particular care to ensure that these prior hypotheses did not lead to positive bias in the results; rather we adopted a predominantly data-led approach, and then compared our end results with the prior hypotheses. In addition to the explicit test of the prior hypotheses suggested by previous recording work, the data-driven approach allows assessment of pitch responses that could not be measured in the previous recording work and comparison with the data from studies based on fMRI.

## Methods

### Subjects

Thirteen healthy volunteers (mean age 23.6, age range 19–47, 5 male), without neurological or hearing impairment, provided informed written consent and participated in the experiment. The study was approved by the National Health Service Research Ethics Committee (ref. 07/Q0905/30).

All subjects completed the experiment without discomfort, excessive head movement of over 10 mm (most subjects moved around 2–3 mm) or any periods of sleep. Two additional subjects completed the experiment (total 15) but their data were not included in the final results; one subject's recording suffered a failure of the MEG head positioning system and therefore could not have their head location co-registered with their MRI scan, and one subject was not able to tolerate the structural MRI scan.

### Stimuli, paradigm and data acquisition

**Fig. 1:**
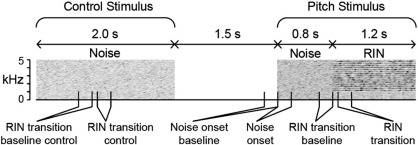
Stimuli. Schematic and time-frequency plot of experimental stimuli. Greyscale plots show time-frequency representation (limited to 5 kHz) of control (left) and pitch (right) stimuli. The RIN component of the pitch stimulus shows the spectral ripples at harmonics of the 256 Hz fundamental, present only above a high-pass filter frequency of 1 kHz. The lower part of the figure shows the key time periods used for analysis, each 230 ms in duration, which are referred to in subsequent figures.

All stimuli were created digitally using Matlab 7.9 software (MathWorks Inc.) at a sampling rate of 44.1 kHz and 16 bit resolution, and presented using Cogent software (http://www.vislab.ucl.ac.uk/cogent.php). Pitch stimuli consisted of 1.2 s of regular interval noise (RIN) preceded immediately by 0.8 s of Gaussian noise. Control stimuli were 2 s of Gaussian noise. RIN was created from Gaussian noise using 16 iterations of an add-same process (Yost et al., 1996) with a delay of 1/256 s. RIN and Gaussian noise were high-pass filtered in the frequency domain from 1 kHz, and the filtered region was filled with fixed-amplitude random-phase noise with a peak power spectral density matched to that of the RIN. Pitch and control stimuli were scaled to the same root-mean-square (RMS) value. The transition from noise to RIN was thus associated with the onset of a pitch percept, but without any change in the gross power spectrum of the stimulus or, therefore, the mean firing rate of auditory nerve fibres. The inclusion of the control stimulus allowed us to examine whether any of the observed results could be explained on the basis of focussed auditory attention or expectation alone, which would be equally present during the relevant part of the control and pitch stimuli. Stimuli were gated with 5 ms onset and offset cosine ramps. Schematics and time-frequency profiles of the stimuli are illustrated in Fig. 1. To maximise power, only the two stimulus conditions described above were used, for each of which 10 exemplars were produced to limit the influence of random peaks in the stimulus waveforms. We acknowledged that this limiting of the experimental stimuli to just two would mean that we could not re-create the previous depth electrode study in its entirety, particularly in assessing responses to RIN above and below the lower limit of pitch, but rather we aimed to obtain proof of concept that equivalent results could be obtained with MEG.

Subjects lay supine, with eyes fixated on a nearby point, while they were passively exposed to the experimental stimuli. Data were acquired for 30 min using a 275 channel whole-head MEG setup with third-order gradiometers (CTF systems) at 2.4 kHz.

Sounds were delivered diotically, via a pneumatic system and Etymotic earmolds, and adjusted to a level comfortable for the subject. 250 repetitions of each stimulus condition (25 per exemplar) were presented in random order, ensuring no immediate repetition of any exemplar, with a fixed inter-stimulus interval of 1.5 s.

A T1-weighted structural MRI scan was acquired at 3 T for each subject (Deichmann et al., 2004).

### Data preprocessing

Data were downsampled to 600 Hz, after preliminary analyses showed that this did not affect results, and converted for analysis in SPM8 (Wellcome Trust Centre for Neuroimaging; Litvak et al., 2011). Preprocessing steps comprised extraction of 3.5 s epochs within timeframe − 0.75–2.75 s with respect to stimulus onset and rejection of any epochs containing peak-–peak amplitude greater than 5 × 10^−12^ T.

### Evoked activity

Average waveforms were computed for all channels, low-pass filtered at 20 Hz and baseline corrected to the 100 ms period immediately preceding stimulus onset or the noise-to-RIN transition. Peaks of interest were taken as the first maximum after each relevant event showing a clearly dipolar scalp distribution. These comprised the noise onset peak, occurring at around 100 ms, and the RIN transition peak, occurring at around 135 ms (Krumbholz et al., 2003). Equivalent current dipoles (ECDs) were fitted to the single time point corresponding to the peak using a variational Bayes equivalent current dipole algorithm (VB-ECD; Kiebel et al., 2008), specifying two independent ECDs for the whole brain with no prior constraints. In all cases there was a clear best ECD in each hemisphere that was reproducible across repetitions of the VB-ECD procedure. Most of the ECDs explained over 95% of the channel domain variance, but in a minority of cases a much poorer fit was obtained, particularly for the noise onset peak. For comparison, ECD fitting was also performed on individual hemisphere data (i.e. half of the channels removed) and yielded consistent results.

### High-frequency oscillatory activity

Single trial sensor data were projected into source space using a beamforming technique implemented in SPM8 and Fieldtrip (Oostenveld et al., 2011). The specific beamformer used was a dynamic imaging of coherent sources (DICS; Gross et al., 2001) method in a vector (as opposed to scalar) implementation. This method constructs an adaptive spatial filter based on the cross-spectral density matrix of the sensor data. We used the real (as opposed to complex) part of the cross-spectral density matrix to derive the filter. The term “adaptive” indicates that the spatial filter is constructed specifically for the given data within a defined time-frequency window. The beamformer produces a brain image, based on a calculation of power at each dipole on a regular 3D grid covering the whole brain volume. We used the average cross-spectral density matrix across trials to construct a single spatial filter, which was then used to create a power image for each individual trial. The filters can also be saved and used to construct “virtual electrode” time series at specified locations. We applied this technique to characterise power changes associated with both the onset of noise and the transition to RIN.

To avoid creating false positive results by performing multiple comparisons, we pre-defined a single time-frequency window for each condition and used this for all subsequent analyses. Based on our prior hypothesis, we initially used a window in the range of 80–120 Hz of 230 ms duration. Also in accordance with our prior hypothesis, we had the window start at 55 ms from the RIN transition, but 0 ms from noise onset. To maximise statistical power, for the baseline window we used the immediate prestimulus period from the same trials. In both cases the baseline window was 230 ms in duration, ending at − 15 ms. This was tested on 6 pilot subjects (not included in final analysis). Based on these pilot results we widened the frequency range to 70–140 Hz, and left the time windows unchanged. To ensure that observed results were not simply the result of selective attention or expectation, we also calculated RIN transition power changes by using the identical time window in the control stimulus as a baseline, instead of the immediate prestimulus period in the RIN condition, which yielded concordant results. The time windows used for analysis are illustrated in Fig. 1.

#### Beamformer source images

For each subject, the beamformer was used to create a normalised image per trial depicting the brain-wide power changes associated with the transition from noise to RIN. A 10 mm grid resolution was used for the analysis. To test statistical significance at the individual level, these images were subjected to a random effects analysis using a one-sample *t*-test. For the group level comparison, each subject's images were averaged to a single image per subject. These were then smoothed using a Gaussian kernel at 12 mm full half-width maximum (FHWM) and subjected to a group random effects analysis with corrections for multiple comparisons across the volume based on random field theory (Friston et al., 1995).

#### Virtual electrode analyses

We determined the spatial location of maximum power in each hemisphere, from the RIN transition period, based on the group analysis. We then used a spatial filter specific to each subject to estimate the virtual electrode time-series data at that location, yielding one time series per subject per hemisphere (total 26). Rather than use the same spatial filters as for the source images (which were adapted to only a short and very specific time window and would hence bias noise cancellation toward this window), we defined a new filter for each subject adapted to both the pitch and control stimuli and to the entire trial length. Each virtual electrode time series was transformed into the time-frequency domain, in EEGlab (Delorme and Makeig, 2004), using a Morlet wavelet analysis covering an extended gamma band from 30–150 Hz, with 6 wavelet cycles at the lowest frequency and 30 at the highest. The average across trials was subtracted prior to this, to yield induced activity. As we used a vector, rather than scalar, implementation of the DICS beamformer, a set of 3 spatial filters was produced for each brain location (corresponding to the three orthogonal dipole orientations). To reduce dimensionality, we performed a principal component analysis (PCA), based on the mean activity across all of time-frequency space for each trial at each orientation, to convert the data for the three orientations into activity for three eigenmodes (corresponding to principal components 1, 2 and 3, in descending order of the amount of variance captured). Activity for each eigenmode was calculated for each point in time-frequency space by matrix multiplying activity across trials and orientations by the weights for the respective principal component. The real part of the data for each eigenmode was then squared to yield a measure of power. The median across trials was taken at each time-frequency point, which has the effect of reducing the influence of residual artefacts. Inspection of the eigenmodes revealed that, for every hemisphere in every subject, the signal of interest existed almost entirely within the third eigenmode, and that the first two eigenmodes contained predominantly noise with little or no discernable signal. Thus the third eigenmode was used for all further analysis. We found that the relative power increases detected with this method were approximately 200 times greater than those obtained by simply averaging the activity from the three dipole orientations (reflecting total power). The RIN transition spectrum was calculated by taking, at each frequency, the mean power within the pre-defined time window of interest (55–285 ms from the RIN transition) and normalising to the mean power across all frequencies within the prestimulus period. We also examined changes in the envelope of 70–140 Hz power, calculated by averaging across frequency at each time point and dividing by spontaneous power (average prestimulus power) within the same band, along the whole trial length. To maximise statistical power, we treated each hemisphere as an individual subject, and combined these into a single group. We also expressed virtual electrode data in the form of the time-frequency domain power values themselves from the pitch condition. Time-frequency domain data were not subjected to a statistical analysis, but time and frequency domain data were compared to the control condition using a series of two-tailed independent samples *t*-tests (p < 0.05 uncorrected).

#### Comparison with direct intracranial recordings

To allow direct comparability with our previous depth electrode recordings of responses to near-identical stimuli – the only differences being a low-pass frequency of 0.8 rather than 1 kHz and durations of noise and RIN of 1 s and 1.5 s respectively – we re-analysed these previous data in keeping with the methods of the present study. For each of the two subjects presented in Griffiths et al., 2010 (subjects 154 and 156), we selected one depth electrode contact per subject located in primary auditory cortex that showed strong and typical responses to noise onset and the RIN transition. The data from 50 repetitions of a pitch stimulus, containing RIN of 256 Hz, per subject were subjected to a Morlet wavelet analysis as described above. We had found, in that study, that responses to RIN occurred across a larger anatomical area than responses to noise. Therefore we did not expect to see the same relative response strengths to RIN and noise for the two modalities, but rather to see qualitatively similar responses.

## Results and discussion

### Evoked activity

**Fig. 2:**
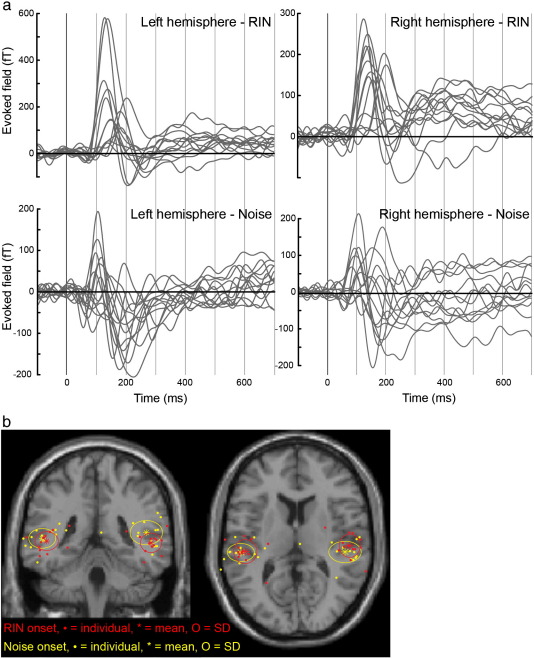
Event-related fields (ERFs). a) ERF waveforms for each subject, shown for each event (noise onset and transition to RIN) and hemisphere. One ERF is shown per subject, taken from the maximally responsive channel to the RIN transition. ERFs are baseline corrected to the 100 ms preceding event onset, and rectified to the predominant direction of field change in response to RIN. b) Source localisations of ERFs. Locations were determined by fitting two equivalent current dipoles (one per hemisphere) to the first maximum following each event that showed a dipolar scalp distribution. All such dipoles are shown on this figure, including the minority for which a satisfactory source reconstruction could not be achieved. Dots represent individual subjects (one per hemisphere), asterisks the group mean and circles the standard deviation. These are displayed in “glass brain” fashion on a template T1-weighted image in MNI space with the coronal and axial sections at y = 0 and z = 0 mm respectively.

All subjects showed bilateral dipolar evoked responses to both the onset of noise and the transition to RIN, as illustrated in Fig. 2. In all but 4 of 52 subject-hemisphere combinations (3 noise, 1 RIN) an ECD explaining over 90% of variance was found. In the remaining cases the fit was less than optimal, but was not excluded from the analysis. The locations of all ECDs are shown in Fig. 2, along with the group mean per hemisphere and the standard deviation. Most ECDs were located in auditory cortex, either HG or PT, the group means were at the border of mid HG and PT, and no consistent difference was seen between the noise onset and RIN transition dipole locations.

### Beamformer source images

**Fig. 3:**
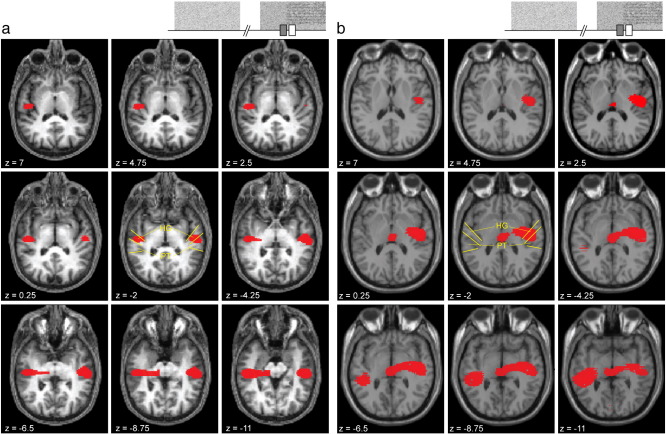
Source reconstructions. Source images for 70–140 Hz power associated with the transition to RIN. Stimulus schematics show the time window used (white rectangle) and the baseline period (grey rectangle). a) Results from a single subject. Red areas show voxels with a significant power increase surviving at a threshold of p < 0.001 uncorrected. b) Group-level results. Red areas show voxels with a significant power increase surviving at a threshold of p < 0.05 corrected to whole brain volume. Slices are axial sections in MNI space tilted in pitch by − 0.5 radians to optimally show Heschl's gyrus (HG) and planum temporale (PT). Z values refer to the vertical distance from the origin in mm. Yellow lines in the central slice highlight the positions of HG and PT.

Fig. 3 shows the areas of power increase in the 70–140 Hz range associated with the transition to RIN from a single subject, displayed on their own MRI scan, and at group-level, displayed on a template T-1 weighted MRI scan. In both these cases, power increases were found bilaterally in auditory cortex, centred at mid-HG and sometimes also medial HG (primary auditory cortex), and extending into PT. Power increases extended beyond auditory cortex, mainly inferiorly and towards the centre of the head, particularly at group level. No power decreases were found. The finding of increased auditory cortex high gamma power is consistent with our prior hypothesis based on depth electrode recordings. Maximal power increase in mid-HG would be expected from the depth electrode results (Griffiths et al., 2010), but could also be expected in other parts of HG or in PT based on previous studies (Griffiths et al., 2001; Patterson et al., 2002; Hall and Plack, 2009). The spatial resolution of beamformer images is dependent on signal to noise ratio (Barnes and Hillebrand, 2003) and, as MEG is much less sensitive to deeper sources, images typically become smoother in deep positions within the head. This is apparent in Fig. 3 where the images appear to extend deeper beyond HG. For this reason it is difficult to say precisely from which areas of auditory cortex the activity derives.

### Virtual electrode analyses

**Fig. 4:**
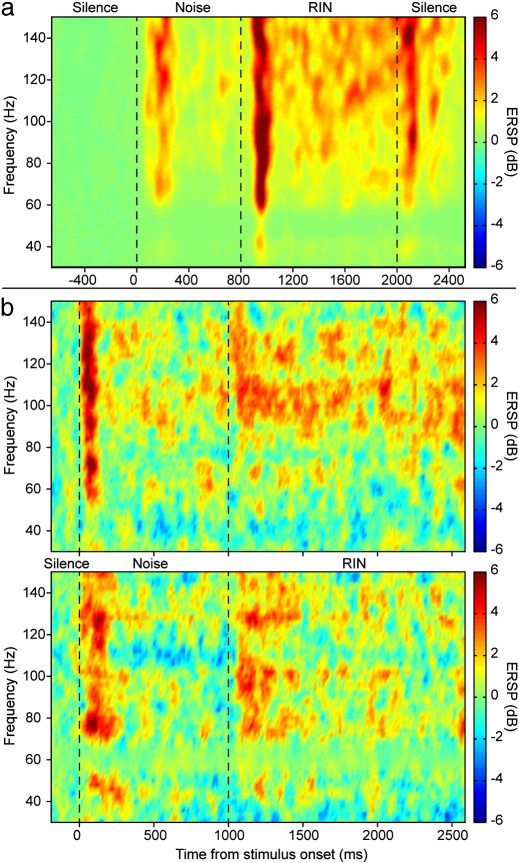
Spectrotemporal decomposition. Time-frequency representations of responses to noise and RIN. a) Group mean data from the present study. b) Individual depth electrode recordings from primary auditory cortex, taken from Griffiths et al., 2010 (subject 154 electrode 1 - upper, and subject 156 electrode 3 - lower). Colour scale represents power change relative to the prestimulus baseline at each frequency, expressed in decibels (dB).

**Fig. 5:**
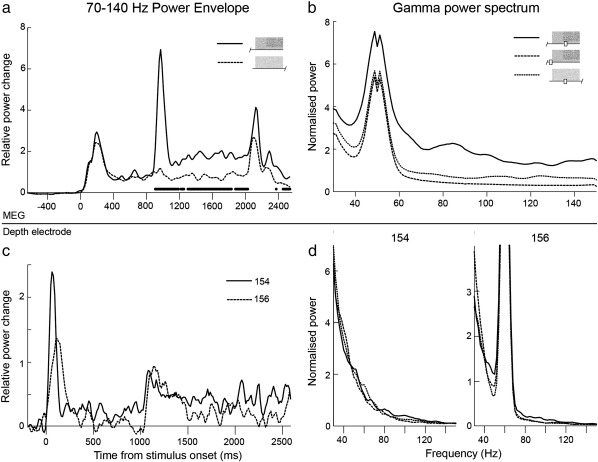
Gamma power spectra and envelopes. Frequency spectra and power envelopes associated with the onset of noise and the transition to RIN. Power change is expressed relative to the baseline period before stimulus onset (i.e. normalised to spontaneous neural activity). a, b) Group level results from MEG virtual electrodes. a) Power envelope for whole stimulus. The solid line indicates the pitch condition and the dashed line the control condition. Noise onset occurs at 0 ms in both stimuli, and RIN onset at 800 ms in the pitch condition only. Dots below the time axis indicate significant power increases (p < 0.05 uncorrected) in the pitch condition over the control condition after the onset of RIN. Stimulus offset occurs at 2000 ms. b) Power spectrum of RIN transition response, with power spectrum during the equivalent part of the control stimulus, and spontaneous (prestimulus) power spectrum shown for comparison. The RIN power spectrum was significantly greater (P < 0.05 uncorrected) at every frequency shown except 50 Hz where electrical noise was present. c, d) Power envelopes and RIN onset power spectra, respectively, from depth electrode recordings. Power changes are calculated as in plots a and b. Solid and dashed lines in c indicate subjects 154 and 156 respectively, and responses to control stimuli are not shown. Fig. 5d is equivalent to b, with one plot for each subject.

Fig. 4 shows the group-level time-frequency representation of responses to all parts of the pitch stimulus. For comparison, equivalent plots are shown for the two subjects' depth electrode recordings from Griffiths et al., 2010. Fig. 5 shows the time-frequency domain data collapsed across either time or frequency, to produce event-related power spectra or power envelopes respectively. Figs. 5a–b illustrate the MEG data and Figs. 5c–d the equivalent depth electrode data. Note that in Figs. 5b and d there is a variable contribution of noise from nearby main electrical circuits, which is at 50 Hz in the UK-acquired MEG data and 60 Hz in the USA-acquired intracortical data. For the MEG data the control stimulus is also shown in the power envelope plots (Fig. 5a, in which gamma responses to noise occur for both stimuli, but responses to RIN occur only with the pitch stimulus) but not for the RIN power spectrum, as this showed no statistically significant changes in any frequency in the control condition. Very similar results are obtained from the MEG and depth electrode recordings, in that there is a rapid and transient response to noise onset of around 200 ms duration, followed by a return to close to baseline power, and then a similar response to the RIN transition, which is followed by a sustained power increase for the duration of the RIN. The two depth electrode subjects show RIN transition spectra which peak around 100 Hz, but with a broader power spectrum for Subject 156 than 154. In the MEG data, the spectral peak occurs at a similar value of around 80–100 Hz, and the spectrum is broad, which would be expected from the superposition of multiple subjects' individual spectra. The MEG power spectrum to RIN was significantly increased, compared to control, at every frequency except at 50 Hz. Response timings show the same pattern for the two modalities, but there is a delay in the order of 25 ms in the response onset times for the MEG data relative to the depth electrode recordings. We excluded delays in the stimulus delivery and recording apparatus as a cause of this discrepancy. Contributing factors could include that neuronal responses need to become synchronised over a larger spatial extent to become detectable with MEG, compared to very localised responses measurable with depth electrodes (i.e. depth electrodes in core auditory cortex would record the true onset of activity, but MEG would only register a power increase once a synchronised power increase had spread to adjacent auditory cortex). The depth electrode data show little sustained power increase due to noise, but a sustained response for the duration of RIN in a similar spectral range to the onset response. These sustained responses were seen in a subset of depth electrodes in primary auditory cortex, while the more lateral electrodes showed transient responses only. In the MEG data, the dominant part of the RIN response is transient, but there is a clear sustained component for the duration of the RIN, before a smaller peak following stimulus offset. In the depth electrode data, in primary auditory cortex, stronger responses are seen to noise onset than the RIN transition, but in the MEG data this trend is reversed. This likely reflects the larger anatomical scale on which pitch is represented compared to noise. Interestingly, the strength of response to noise onset is similar in the two modalities, but the transient and sustained responses to RIN are much stronger in the MEG data. This seems counter-intuitive initially, but could potentially be explained if either 1) The depth electrode contacts were not placed at the maximally responsive locations to RIN, or 2) The DICS spatial filtering and PCA methods used in the MEG analysis provide cancellation not only of noise from sources outside the brain region of interest but also of noise within that brain region.

The broad consistency of the spectrotemporal profiles between the recording modalities lends strong support to the validity of the MEG results in their relevance to true auditory cortical oscillations in the high gamma range.

## Conclusions

Using non-invasive neuroimaging, our present results corroborate the finding that auditory cortical oscillations in the high gamma range (above 60 Hz) are present in response to the transition from Gaussian noise to RIN, which has previously only been demonstrated in two human subjects with implanted electrodes. We acknowledge that the MEG results in isolation do not distinguish these oscillations as a correlate of pitch as opposed to a more generic response to certain structured auditory stimuli. However the previous depth electrode study used a range of RIN frequencies and found that the gamma response at the RIN transition was only present when that RIN was of a frequency associated with the perception of pitch. Our argument for the relevance of the MEG results as a correlate of pitch perception is based on this trend in the depth electrode results, and on the strong similarity of the results obtained from the two methods. Subsequent research should continue to test this assertion, along with the role of other potentially influential factors such as the role of selective attention, which has been shown to modulate both early (Hari et al., 1989) and late (Rif et al., 1991) auditory cortical evoked fields, and also auditory high gamma oscillations (Ray et al., 2008).

Our results provide proof of principle of an approach, to non-invasively study an important but low SNR correlate of perception, which can be adopted for future studies on pitch or other percepts. Interestingly, the MEG method was able to capture power changes of the same relative magnitude as in depth electrode recordings. The strong concordance of these results, in the spatial and spectrotemporal domains, with the gold standard technique of depth electrode recording confirms the validity of this method to obtain a meaningful and accurate representation of high-frequency activity within the auditory cortex. This will be especially important for future studies where such gold standard results are not available.
